# Exposure to *Candida albicans* Polarizes a T-Cell Driven Arthritis Model towards Th17 Responses, Resulting in a More Destructive Arthritis

**DOI:** 10.1371/journal.pone.0038889

**Published:** 2012-06-12

**Authors:** Renoud J. Marijnissen, Marije I. Koenders, Frank L. van de Veerdonk, John Dulos, Mihai G. Netea, Annemieke M.H. Boots, Leo A.B. Joosten, Wim B. van den Berg

**Affiliations:** 1 Rheumatology Research and Advanced Therapeutics, Department of Rheumatology, Radboud University Nijmegen Medical Centre, Nijmegen, The Netherlands; 2 Department of Medicine and Nijmegen Institute for Infection, Inflammation and Immunity (N4i), Radboud University Nijmegen Medical Centre, Nijmegen, The Netherlands; 3 Department I&DL, Section Pharmacology, Crucell, Leiden, The Netherlands; 4 Department of Rheumatology and Clinical Immunology, University Medical Centre Groningen, University of Groningen, The Netherlands; University of Southern California, United States of America

## Abstract

**Background:**

Fungal components have been shown very effective in generating Th17 responses. We investigated whether exposure to a minute amount of *C. albicans* in the arthritic joint altered the local cytokine environment, leading to enhanced Th17 expansion and resulting in a more destructive arthritis.

**Methodology:**

Chronic SCW arthritis was induced by repeated injection with *Streptococcus pyogenes* (SCW) cell wall fragments into the knee joint of C57Bl/6 mice, alone or in combination with the yeast of *C. albican*s or Zymosan A. During the chronic phase of the arthritis, the cytokine levels, mRNA expression and histopathological analysis of the joints were performed. To investigate the phenotype of the IL-17 producing T-cells, synovial cells were isolated and analyzed by flowcytometry.

**Principal Findings:**

Intra-articular injection of either Zymosan A or *C. albicans* on top of the SCW injection both resulted in enhanced joint swelling and inflammation compared to the normal SCW group. However, only the addition of *C. albicans* during SCW arthritis resulted in severe chondrocyte death and enhanced destruction of cartilage and bone. Additionally, exposure to *C. albicans* led to increased IL-17 in the arthritic joint, which was accompanied by an increased synovial mRNA expression of T-bet and RORγT. Moreover, the *C. albicans*-injected mice had significantly more Th17 cells in the synovium, of which a large population also produced IFN-γ.

**Conclusion:**

This study clearly shows that minute amounts of fungal components, like *C. albicans*, are very potent in interfering with the local cytokine environment in an arthritic joint, thereby polarizing arthritis towards a more destructive phenotype.

## Introduction

Rheumatoid arthritis (RA) is a systemic joint disease with an unknown etiology, characterized by a chronic inflammatory infiltration of the synovial membrane, and associated with destruction of cartilage and bone. Both genetic and environmental factors contribute to the development of disease. Various infectious agents, such as bacteria and viruses have long been associated with the pathogenesis of RA [1], [2]. The involvement of these micro-organisms has not only been proposed in the initiation of arthritis, but also in the progression and exacerbations of the disease.

Microorganisms are recognized by pattern recognition receptors (PRR), such as Toll-like receptors (TLRs) and C-type lectins, which are essential components of the innate immune system and form the bridge with adaptive immunity. If the inflammatory response is not adequate and/or the infection is not cleared properly, persistence can eventually result in chronic or even auto-immune inflammation [2]. Particularly TLR signaling has been shown to play an intrinsic role in the inflammatory processes of arthritis [3]–[5]. The concept that commensal pathogens affect the pathogenesis of RA is strengthened by data from spontaneous murine arthritis models and the notion that spontaneous arthritis does not develop in animals kept under germ free conditions [3], [6]. This implies the importance of (memory) responses for the ‘break of tolerance’ and induction of auto-inflammation/immunity.

*Candida albicans* is the most common opportunistic fungal pathogen in humans. Infection with *C. albicans* induces IL-17 producing T helper (Th17) cells *in vitro* and *in vivo* in naïve mice [7]–[9]. Under physiological conditions, these Th17 cells produce proinflammatory cytokines like IL-17A (IL-17), IL-17F, IL-21 and IL-22, and are involved in the clearance of several extracellular bacteria and fungi [10]. In the arthritic joint, direct or indirect effects of IL-17/Th17 result in increased inflammation, angiogenesis, and osteoclastogenesis, resulting in enhanced breakdown of cartilage and bone [11]–[14]. Although *C. albicans* or *Candida*-derived β-glucan have been shown to induce and aggravate various models of arthritis [15], [16], these observations have not yet been linked to modulation of the IL-17/Th17 pathway and increased structural damage.

The purpose of the present study was to demonstrate the ability of *C. albicans* to skew the T-cell balance in the chronic murine SCW model. This model initiates as a local TNF-dependent macrophage-driven inflammation, at which repeated antigen exposure results in a chronic T-cell dependent arthritic process [17]. A small quantity of *C. albicans* or Zymosan A (<10% of mass) was added to the cell wall fragments of *Streptococcus pyogenes* (SCW) that were repeatedly injected into the knee joint. During the chronic phase of the arthritis, the development of macroscopic joint swelling and histopathological changes in synovium, cartilage, and bone were determined. Furthermore, the levels of antibodies, secretion of T-cell cytokines and presence of T-cells were examined.

## Materials and Methods

### Animals

Male C57Bl/6 mice were purchased from Janvier, France. The mice were housed in filter-top cages; water and food were provided *ad libitum*. The mice used were between 10–12 weeks of age. All animal procedures were approved by the animal ethics committee of the Radboud University Nijmegen.

### Study protocol

*Steptococcus pyogenes* T12 organisms were cultured and prepared as described previously [17]. For the fungal components, the blastoconidia of *Candida albicans* (ATCC MYA-3573 (UC 820)) were used [8]. Zymosan A (*Saccharomyces Cerevisiae*) was purchased from Sigma-Aldrich and prepared as described earlier [18]. Chronic unilateral arthritis was induced by repeated intra-articular (i.a.) injection of 25 µg SCW on days 0, 7, 14, and 21, whether or not with 1*10^5^ particles of *C. albicans* (1*10^5^≈1 µg) or 2 µg Zymosan, in 7 µl phosphate buffered saline (PBS) into the right knee joint of naive mice. As a control, additional groups were injected with the fungal particles alone. On day 22, twenty-four hours after the last injection, a subgroup of mice was sacrificed for the collection of synovial washouts. Accordingly, patellae with surrounding soft tissue were isolated from inflamed knee joints and cultured 1 hour at RT in RPMI-1640 medium containing 0.1% BSA (200 µl/patella). In addition, the draining lymph nodes (popliteal and inguinal) were collected and cells were isolated. Then, 1*10^5^ cells were stimulated for 72 hours with 2 µg/ml plate bound anti-CD3 (R&D systems) and 2 µg/ml plate bound anti-CD28 (BD Biosciences). Thereafter, supernatants were collected, centrifuged and stored for cytokine determination. On day 28, during the chronic joint inflammation, the sera from the remaining mice were collected, the mice were sacrificed, and knee joints were prepared for histology.

### Measurement of joint swelling

Joint swelling was assessed by measuring the accumulation of ^99 m^Tc in the inflamed joint due to increased blood flow and edema. Therefore, 0.74 MBq of ^99 m^Tc in 200 µl of saline was injected subcutaneously. After several minutes of distribution throughout the body, external gamma radiation in the knee joints was measured. Swelling was expressed as the ratio of gamma counts in the right (inflamed) knee joint to gamma counts in the left (control) knee joint. Values higher than 1.1 counts per minute were considered to represent joint swelling.

### Histopathology

For standard histological assessment, the isolated joints were fixed for 4 days in 10% formalin, decalcified in 5% formic acid, and the specimens were processed for paraffin embedding. Tissue sections were stained with hematoxylin and eosin. The severity of inflammation in the joints was scored on a scale of 0–3 (0=no cells, 1=mild cellularity, 2=moderate cellularity, and 3=maximal cellularity). Bone destruction was graded on a scale of 0–3, ranging from no damage to the complete loss of bone structure. Proteoglycan (PG) depletion was determined using Safranin O staining. Loss of proteoglycans was scored on a scale of 0–3, ranging from normal, fully stained cartilage to destained cartilage, fully depleted of PG. The scoring of the sections was performed in a blinded manner.

### Cytokine detection

Cytokine levels were measured using Luminex multi-analyte technology in combination with Bio-Plex cytokine kits (Bio-Rad; IL-17, IL-4, IFN-γ, IL-10) and performed according to manufacturer's instructions.

### Quantitative PCR

RNA was isolated from synovial knee biopsies using TRI-reagent (Sigma) and treated with DNase to remove genomic DNA. The RNA was subsequently reverse transcribed with oligo(dT) primers in a reverse transcriptase procedure. Quantitative real-time PCR was performed with cDNA specific primers (Biolegio) and SYBR Green PCR Master Mix (Applied Biosystems). The ΔCt method was used to normalize transcripts to GAPDH and to calculate relative mRNA expression (2^−ΔCt^), for which the expression in the SCW injected group was arbitrarily set to 1. All primer pairs were developed using Primer Express 2.0 (Applied Biosystems) and validated according to protocol.

### Isolation and stimulation of synovial cells

After sacrificing the mice, the ankle joint synovium was dissected for single cell isolation. In short, synovial biopsies were incubated with enzymatic digestion buffers (Liberase Blendzyme, Roche) for 30 minutes at 37°C. Next, a 70 µm nylon cell strainer (BD Falcon) was used to process the digested tissue. The cell preparation was collected in RPMI with 10% FCS. To isolate the single mononuclear cells, Lympholyte-M (Cederlane) was used according to manufacturer's protocol. All cells were cultured in RPMI-1640 (Gibco; Invitrogen) supplemented with 10% FCS. Subsequently, the cells were prepared for intracellular flowcytometry.

### Flow cytometry

Synovial cells were stimulated for 5 hours with PMA (50 ng/ml; Sigma) and ionomycine (1 µg/ml; Sigma) in the presence of Golgiplug (BD Biosciences) according to manufacturers protocol. After staining the cells extracellularly with anti-CD3 APC (BD Biosciences), the cells were fixed and permeabilized with Cytofix/Cytoperm solution (BD Biosciences). Subsequently, they were intracellularly stained with anti-IFN-γ PE (BD Biosciences) and anti-IL-17 FITC (BD Biosciences). Samples were measured on a FACS Calibur and data were analyzed using FlowJo software.

### Determination of anti-SCW antibody levels

Levels of anti-SCW antibodies in the serum of mice with chronic SCW-induced arthritis (day 28) were analyzed according to standard methods [17]. Briefly, 10 ng of SCW fragments were coated overnight onto 96-well plates. Thereafter, plates were washed, and nonspecific binding was blocked with 1% BSA in PBS-Tween 80 (0.05%). Anti-SCW antibodies were examined in serial 2× dilutions, starting with an initial dilution of 20×. After incubation for 1 hour, plates were washed and isotype-specific goat anti-mouse Ig–HRP (1∶1,000) was added for 1 hour at room temperature.

### Statistical analysis

Results are expressed as the mean ± SEM. Differences between experimental groups were tested using Mann-Whitney U-test or one-way analysis of variance with Dunnett's multiple comparison test, as appropriate. *P*-values less than 0.05 were considered significant.

## Results

### Exposure to fungal particles aggravates chronic SCW arthritis

Repeated intra-articular injection of SCW bacterial fragments induce arthritic flares that shift from a predominantly macrophage-driven acute inflammation to a T-cell driven chronic inflammation [17]. To investigate the ability of *C. albicans* to effectively alter the local synovial inflammatory process, heat-killed conidia of *C.albicans* were co-injected with 25 µg SCW fragments in the knee joint on day 0, 7, 14 and 21. In preliminary experiments, exposure to differential doses of C. albicans to murine peritoneal macrophages resulted in the production of various innate cytokines like TNF-α, IL-1β and IL-6 (figure S1). Subsequently, during a pilot experiment, we selected a dose of 1*10^4^ conidia of *C. albicans*, which we co-injected with the SCW fragments into the knee joint. This dose did not show a significant alteration in arthritis severity/score (data not shown). We therefore increased the dose of C. Albicans to 1*10^5^ on top of the SCW dose of 25 µg. For comparison, we injected 2 µg Zymosan A, a glucan derived from the yeast cell wall of *Saccharomyces Cerevisiae*, known for its adjuvant properties [19]–[21]. As control, mice were injected i.a. with SCW, *C. albicans* or Zymosan A alone.

**Figure 1:**
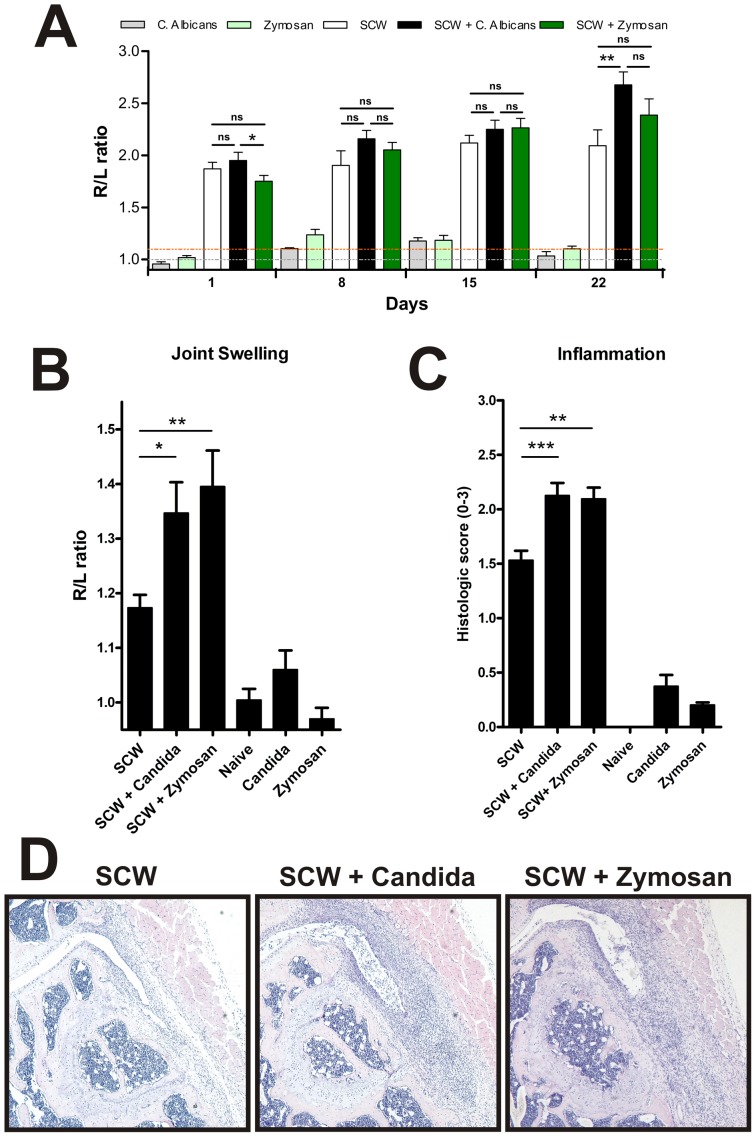
*Candida albicans* and Zymosan A aggravate the inflammation in the chronic SCW model. On days 0, 7, 14, and 21, streptococcal cell wall (SCW) fragments were injected intra-articularly (i.a.) into the knee joint. Joint swelling (ratio of ^99 m^Tc uptake in the treated right knee joint to that in the untreated left knee joint) were determined 1 day after every injection (A) and during the chronic phase of the model on day 28 (B; n=6 mice/group). In addition, the inflammatory cell influx (C) on histological slides was quantified on day 28. H&E stained frontal knee sections (original magnification 100×) (D) are shown (n=8 mice/group. Values are the mean ± SEM; ns=non significant, * P<0.05, ** P<0.01, *** P<0.001, by One-way ANOVA.

The control mice injected with *C. albicans* or Zymosan A alone did not develop joint swelling after the injections (figure 1A). Furthermore, the co-injection of *C. Albicans* or Zymosan A on top of the SCW fragments did not result in an increased joint swelling compared to the SCW injected group during the acute phase of the model. On the contrary, one day after the final injection, on day 22, the *C. Albicans* co-injected group showed a significant increase in the technetium measurements, whereas the Zymosan A co-injected group only showed a trend for increase. Accordingly, we analyzed the joint swelling and histology during the chronic phase of the model, on day 28. During this phase, the joint swelling in the mice injected with SCW remained significantly increased (figure 1B). Interestingly, exposure to minute amounts of *C. albicans* or Zymosan A on top of the SCW fragments significantly aggravated the joint swelling. This increase in joint swelling in both the *C. albicans* and Zymosan A injected mice was supported by a significant increase in the influx of inflammatory cells after histopathological analysis (figure 1 panels C and D), indicating that both fungal particles aggravated the inflammatory process during the chronic phase of the model.

### Increased joint destruction in SCW arthritis after co-exposure to C. albicans

**Figure 2:**
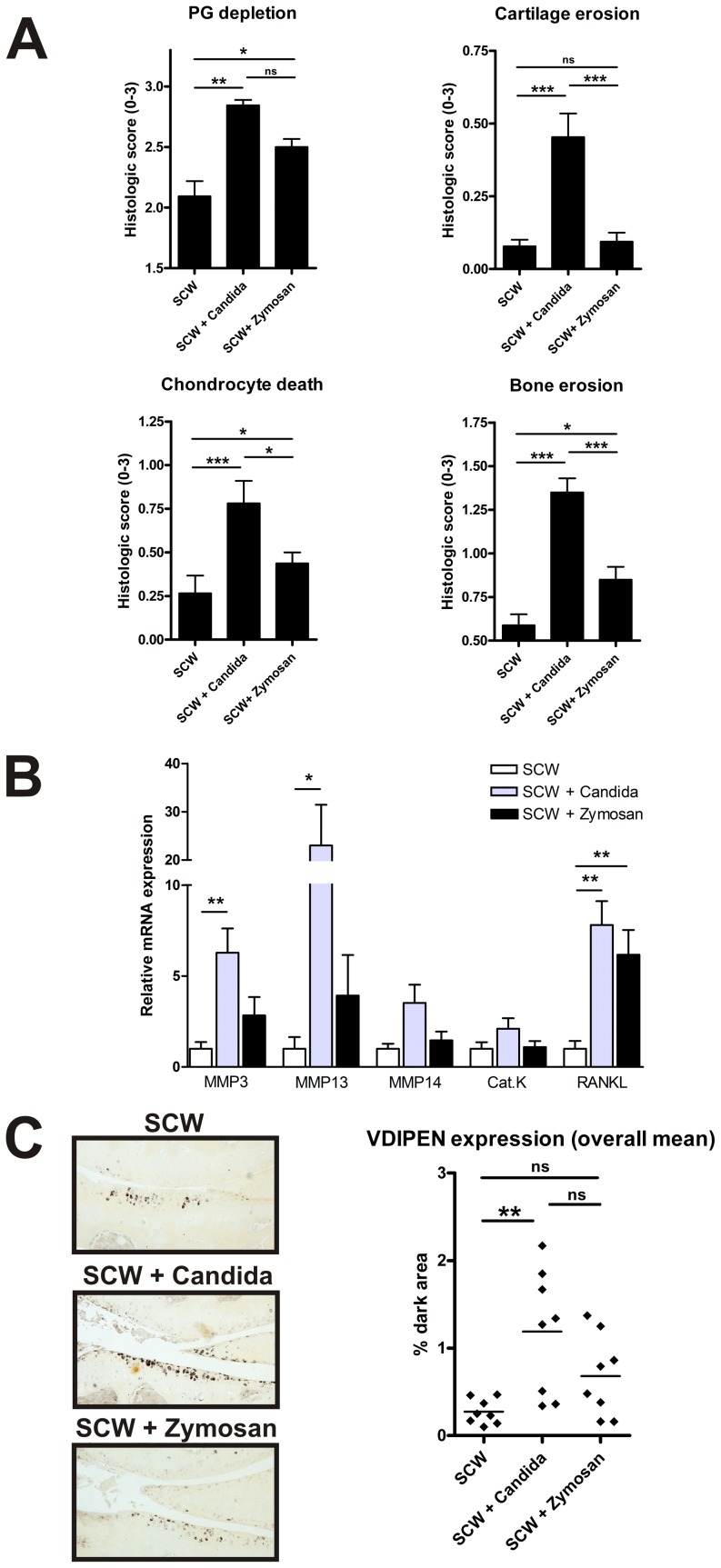
Exposure to *C. albicans* increases the cartilage destruction and bone erosion during chronic SCW arthritis. Analysis of destructive parameters after 4 relapsing flares of arthritis. On day 28, knee joints (n=8/group) were harvested for histological assessment. Knee joint sections were stained using Safranin O to determine the degree of proteoglycan (PG) depletion. H&E staining was used to score the degree of chondrocyte death, cartilage surface erosion and bone erosion (A). QPCR analysis of destructive related genes was performed on synovial biopsies (n=3/group) of day 22, one day after the last injection (B). Representative images of arthritic knee joints showing immunohistochemical staining for VDIPEN after the 4 repeated injections (day 28) (C). Besides, the quantitative measurement of VDIPEN expression (percentage of positively stained area) in the cartilage of the 3 groups of mice (n=8/group) was analyzed. Values are the mean ± SEM; ns=non significant * P<0.05, ** P<0.01, *** P<0.001, by One-way ANOVA.

To compare the ability of *C. albicans* and Zymosan A to affect the cartilage and bone destruction, we performed a detailed histological analysis. Compared to the SCW-injected mice, the increased joint inflammation at day 28 after co-injection of SCW with fungal particles was accompanied by marked proteoglycan depletion (figure 2A). Remarkably, although the influx of inflammatory cells was comparable after addition of either *C. albicans* or Zymosan A, we observed a significantly higher increase in cartilage erosion, chondrocyte death and bone erosion in the *C. albicans* co-injected group compared to the SCW injected group (figure 2A), suggesting that Candida exposure modulates towards a more destructive immune response.

In addition, we analyzed the mRNA expression of several inflammatory genes involved in bone and cartilage destruction using synovial tissue collected during the chronic phase of the arthritis. While the osteoclast marker Cathepsin K (Cat.K) was not significantly enhanced, RANKL was upregulated in the synovium in both the *C. albicans* and Zymosan A induced groups, in line with to the increase in bone erosion. Moreover, the matrix metalloproteinase's MMP3 and MMP13 were significantly increased in the synovium of *C. albicans* inoculated mice compared to the single SCW group, corresponding to the increased cartilage erosion (figure 2B).

To support that the increase in cartilage destruction was induced by a matrix metalloproteinase–mediated process, we examined the expression of the MMP-specific aggrecan neo-epitope VDIPEN by immunohistochemistry. Repeated SCW injections were sufficient to induce VDIPEN expression at the cartilage sites (figure 2C). The expression of VDIPEN after four weeks of SCW arthritis was comparable to the Zymosan A co-injected mice. However, in line with the increase in MMP mRNA expression, the amount of VDIPEN expression increased dramatically when *C. albicans* had been co-injected with SCW into the joints. These findings show that a small amount of *C. albicans* is sufficient to exacerbate the chronic murine SCW arthritis model by increasing the cartilage and bone destruction.

### Exposure to *C. albicans* during chronic SCW arthritis skews the T cell cytokine profile towards Th17 responses

**Figure 3:**
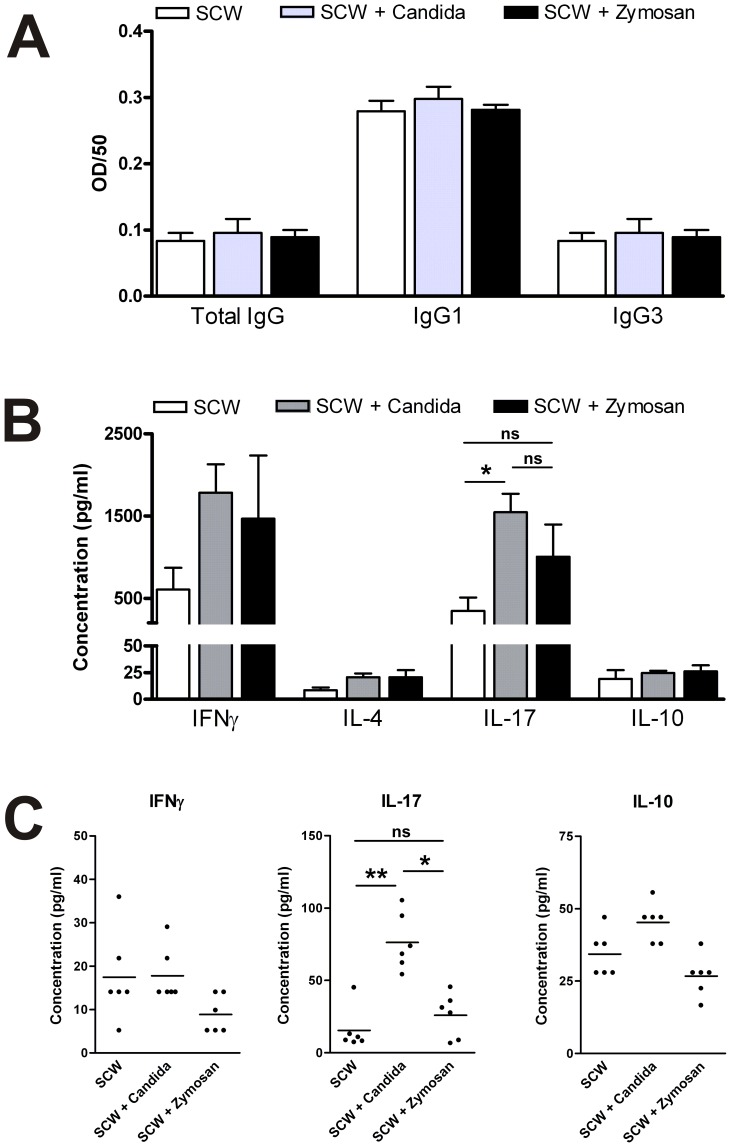
*C. albicans* skews T-cell cytokine profile. Serum levels of anti-SCW-specific total IgG, IgG1, and IgG3 antibodies (A). Draining lymph node cells (1*10^5^/well) were stimulated with anti-CD3/anti-CD28 antibodies for 72 hours (n=6/group). Levels of IFN-γ, IL-17 and IL-10 were determined by Luminex in the supernatants of the stimulated lymph node cells (B) and washouts of synovial biopsies of day 22 (C). Results are the mean ± SEM; ns=non significant *=P<0.05; **=P<0.01, ***=P<0.001, by One-Way ANOVA.

To study modification of the adaptive immune responses, we initially studied the B-cell compartment and determined the levels of anti-SCW antibodies of total IgG, IgG1 and IgG3 in the sera of the mice. We previously demonstrated that in the chronic SCW model, antigen-specific antibodies are induced after the fourth i.a. injection of SCW fragments [22]. As can be appreciated from figure 3A, we observed no differences in the serum levels of SCW specific antibodies between the groups, indicating that the B-cell antibody production was not altered after addition of *C. albicans* or Zymosan A.

Since T helper cells are crucial for the chronic phase of the SCW arthritis model, and Candida is known for the induction of Th17 cells, we explored alterations in T-cell responses. Pan T-cell stimulation with anti-CD3 and anti-CD28 of draining lymph node cells on day 22 revealed no difference in the levels of IL-4 and IL-10 production. Interestingly, a significant increase in IL-17 production and a tendency for increased IFN-γ production were found, suggesting an enhanced helper T cell response of mainly the Th17 lineage by the addition of *C. albicans* (figure 3B).

To conclude whether this peripheral increase in IL-17 and IFN-γ represented a shift in the local T-cell balance in the arthritic joint, we measured the same T-cell derived cytokines in the synovial washouts of the mice. IFN-γ and IL-10 levels were not altered, and IL-4 was undetectable (figure 3C). In contrast, the IL-17 levels were significantly increased in the *C. albicans* co-injected group, while the cytokine levels in the Zymosan A co-injected group were not different from the SCW group. All together, these data suggest that *C. albicans* induces a shift in the T cell compartment during SCW arthritis, favoring Th17.

### *C. albicans* mainly induces IL-17-producing T cells in the joint

**Figure 4:**
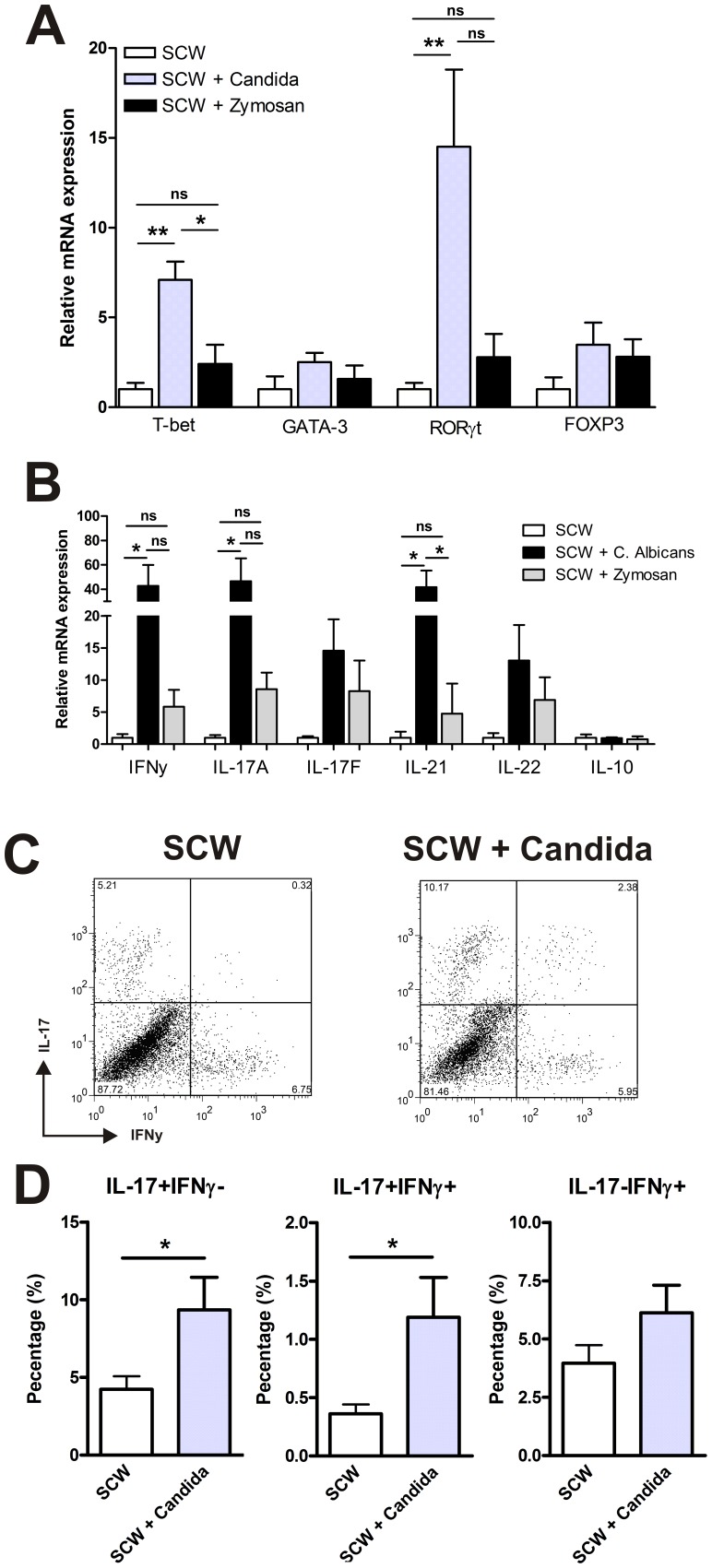
Co-exposure to *C. albicans* induces Th17 cells in the joint. The mRNA expression of T-bet, GATA-3, RORγT and FOXP3 in synovial biopsies of the knee joints (n=3 mice/group) were measured by QPCR (A) The mRNA expression of IFNy, IL-17A, IL-17F, IL-21, IL-22 and IL-10 are shown (B). The knee joint synovium was dissected and prepared for enzymatic digestion. The cells were incubated with PMA/ionomycine and Golgiplug for 5 hours before flowcytometric analysis (n=6 mice/group) and stained for CD3, IL-17, and IFN-γ. Representative dot plots of the isolated cells gated on CD3+ are shown (C). The mean percentage of isolated IFN-γ and IL-17 producing T cells (CD3+ population) are shown (D). Results are mean ± SEM; ns=non significant *=P<0.05, **=P<0.01, by one-way ANOVA.

To establish a shift in the T-cell compartment, we subsequently determined the levels of T-cell transcriptional lineage factors, which specify the different T helper cell lineages. FOXP3 (Treg) and GATA3 (Th2) mRNA expression levels were not significantly different between the SCW-arthritis groups (figure 4A). In line with the enhanced IL-17 production from the lymph nodes, we observed a significant increase in the mRNA expression of the Th17 transcription factor RORγT after *C. albicans* injection. Remarkably, although the level of IFN-γ was not significantly increased in the synovial washouts, we did observe an increase in Th1 transcriptional lineage factor T-bet. Co-exposure to Zymosan A did not influence the other transcription factors. To further confirm the Th17 profile, we analyzed the expression of IL-17A, IL-17F, IL-21, IL-22 and IFNy (figure 4B). Here we again observed a significant increase in the expression of IL-17A, IL-21 and IFNy. Although a trend for an increase was found for the other Th17 profile cytokines, they were not significantly increased in the *C. albicans* co-injected group compared to the SCW and SCW+Zymosan A groups.

Next, we determined whether the inoculation of *C. albicans* in the chronic SCW model affected the phenotype of the infiltrated T-cells. On day 22, at the peak of the inflammation, we dissected the inflamed synovium and isolated the residing cells. Intracellular cytokine staining revealed an increased percentage of single IL-17 producing T-cells (figure 4B, C). Further analysis revealed that a subpopulation of these IL-17 producing T cells, also produced IFN-γ, partly explaining the increase in the Th1 transcription factor T-bet. This indicates that in the presence of *C. albicans* the T-cell balance during SCW-arthritis is shifted towards a pronounced Th17/Th1 profile.

## Discussion

Our main finding is that a low-grade exposure to *C. albicans* can skew the T cell balance in the SCW arthritis model, by inducing Th17 cells and pushing it towards a more destructive arthritis model. This indicates that the presence of a minute amount of *C. albicans* antigen, after *e.g.* a mucosal infection, might be sufficient to skew an inflammatory cytokine profile.

Microbial infections have long been associated with the pathogenesis of RA. Therefore, several fungi and fungal-derived ligands have been used to provoke or accelerate arthritic processes. In the last decade, several PRR's that recognize fungal PAMPs have been identified, in particular Toll-like receptors (TLR-2 and TLR-4) and C-type lectin family members (Dectin-1, Dectin-2 and mannose receptor) [6], [15], [16], [18], [20], [23]. *C. albicans* is especially known for the induction of Th17 cells via Dectin-1, TLR-2, TLR4, Mannose receptor, and complement [8], [24] Earlier, it has been shown that gut colonization by *C. albicans* or exposure to *C. albicans* derived β-glucans can initiate and aggravate collagen-induced arthritis, probably via an increased and/or altered cytokine profile [16], [25]. Although this shows that *C. albicans* can initiate and aggravate arthritis models, it was not known whether and how Th17 responses were mechanistically involved.

To explore the potency of a minute amount of *C. albicans* on skewing T-cell responses during arthritis, we investigated the chronic SCW model. In this model, chronic T cell dependent arthritis is induced by repetitive intra-articular injection of *Steptococcus pyogenes* cell wall (SCW) fragments [17]. *S. pyogenes* is a common Gram-positive bacteria that can induce severe rheumatic fever and/or reactive arthritis, depending on the host response [26]. Previously, we have shown that intra-articular exposure to SCW mainly depends on macrophage activation via TLR-2/MyD88 pathway [27]. Repeated exposure results in a chronic arthritis involving SCW-specific T- and B-cell memory responses, without the need for adjuvants like Freund's complete adjuvant. Furthermore, although the model involves a chronic phase that is characterized by an IL-1 and IL-17 dependent cartilage destruction, the model is less destructive compared to collagen-induced arthritis (CIA) and hardly involves bone erosions. We hypothesized that co-exposure to *C. albicans*, would alter the macrophage-derived cytokine production during the flares, aggravating the chronic disease.

Monocytes and macrophages stimulated with *C. albicans* induce pro-inflammatory cytokine production, like TNF-α, IL-1 and IL-6 [24]. To prevent an aggravation of the SCW-induced arthritis by the addition of Candida, we choose the dose of C. Albicans at such a subtle level that it did not cause a significant increase in the joint swelling on top of the S. pyogenes cell wall (SCW) induced local inflammation. During this acute phase of the SCW arthritis model, joint swelling is TNF-α dependent [28]. We therefore conclude that a possible Candida-induced increase in TNF-α could not be observed by our TNF-dependent Tc-measurement. Even three weeks of repeated co-injections with *C. albicans* or Zymosan did not significantly accelerate the model, showing that the co-exposure to *C. albicans* did not increase the innate immune response like a classical adjuvant [29], but may have prolonged or altered the immune response.

This was further strengthened by the fact that we observed no changes in classes of SCW-specific immunoglobulin production. These SCW specific antibodies can be detected in sera during the late chronic stage, and are also TNF-α dependent [17]. Interestingly, B-cells were reported to be involved in the Th17 response after *C. albicans* exposure [30]. And although B-cells play a minor role in the repeated SCW arthritis model [17], co-exposure to *C. albicans* might have altered the antigen presenting function of B-cells. The presence of autoantibodies, like rheumatoid factor (RF) and anti-citrullinated petide antibodies (ACPA) in the sera of patients are early predictors of disease and correlate with arthritis severity [31].

At day 22, one day after the last injection, when the model shifts from a predominately macrophage driven model to a T cell dependent model, we observed a significantly increased joint swelling in the Candida co-injected group, with a trend for increase in the Zymosan co-injected mice. Furthermore, during the late chronic phase of the model (at day 28) we observed a prolonged increase in joint swelling after co-exposure to both *C. albicans* or Zymosan A. Interestingly, although the increase in joint swelling and inflammatory cell influx in the *C. albicans* co-injected mice was comparable to Zymosan A co-injected mice, the chondrocyte death, bone erosion and cartilage erosion were significantly elevated by *C. albicans*. Additional analysis revealed that during the chronic phase of the model, the *C. albicans* exposure contributed to the increased MMP-mediated cartilage destruction and increased bone erosion, suggesting that additional processes were involved in the *C. albicans* exposed joints. This increase in joint destruction was accompanied by local synovial IL-17 expression, which is thought to/may enhance the role of TNF-α in the progression of the destructive process [32]–[34]. Synergy between IL-17 and TNF-α may underlie the increased pathology in the *C. albicans* co-injected group. Together, we concluded that the low levels of *C. albicans* and Zymosan A did not accelerate the model during the first three weeks of repeated antigen injections, but addition of *C. albicans* did alter the T cell balance and arthritis outcome at the end of the experiment.

Focusing on the T-cell cytokine production, we observed an increased Th17/Th1 cytokine profile. In line with the increase in the transcriptional factors T-bet and RORγT, this increase was related to an increase in IL-17 single and double positive T-cells. Furthermore, over 90% of the total IL-17 producing cells were CD3+ (data not shown). This supports that T lymphocytes are the main source of IL-17 in this model, rather than innate immune cells capable of producing IL-17 [35].

T helper cells are defined T lymphocytes expressing CD4 glycoprotein on their cell surface. We chose CD3 staining over CD4 staining due to the reported sensitivity of CD4 to cleavage during enzymatic digestion of the studied tissue [36]. Only recently, we have altered the protocol of enzymatic digestion slightly, to prevent CD4 cleavage. In a yet unpublished observation (submitted manuscript) we observed that in the SCW model more than 80% of the IL-17+ producing cells express CD4.

Apparently, *C. albicans* does not only increase the ‘classical’ IL-17 producing T-cells, but also promotes the transition to a Th1/Th17 mixed phenotype. The role and induction of Th1 profile versus Th17 profiles in models have received increased attention. In human RA, IFN-γ producing T helper cells are found at the level of the synovial tissue rather than IL-17 producing T helper cells [37], [38], whereas the experimental arthritis models do illustrate an important role for IL-17 [14]. The strength of the IFN-γ response seems to determine the dependency on IFN-γ or IL-17 [39]. Remarkably, isolated IFN-γ producing T helper cells from patients with chronic inflamed joints show more resemblance with ‘classical’ Th17 cell surface repertoire compared to ‘classical’ Th1 cells [40], [41]. In addition, recent reports have shown the plasticity of Th17 cells *in vivo*, that shift towards IFN-γ producing cells during chronic autoimmune inflammation [42], [43]. Both IL-12 and IL-23 might be important players for this transition [41], [42], [44].

On the other hand, recent studies have shown an inverse relationship between Th17 cells and regulatory T cells [45]. Tregs in the joints of RA patients can inhibit the production of IFN-γ and IL-17 [46]. Although we did not observe a significant alteration in the FOXP3 levels, the increased IL-6 levels after C. albicans exposure, might contribute to the imbalance between Th1/Th17 and Tregs cells [47]. These results raise questions about the ancestry and transcriptional control of Th17 during the course of chronic inflammatory diseases, such as Rheumatoid Arthritis. Clarifying pathways that control the induction of these double positive cells *in vivo* during arthritis will lead to new strategies for the development of novel therapeutic interventions in the Rheumatic diseases.

In conclusion, exposure to a small amount of the fungal pathogen *C. albicans* is sufficient to shift the T-cell balance of the synovial immune response towards a more pronounced Th17/Th1 profile, thereby aggravating the model towards increased cartilage and bone destruction. In the past others have shown that fungal particles, like *C. albicans*, can accelerate or aggravate arthritic disease. This is the first report showing that this aggravation involves the induction of Th17 cells *in vivo*. *C. albicans* is a common commensal that can cause mucosal and systemic infections [48]. Whether *C. albicans* can contribute to the progression of joint destruction in RA remains to be elucidated.

## Supporting Information

Figure S1 ***Candida albicans*** **and Zymosan A induce a different cytokine profile in vitro.** 1*10^5^ peritoneal macrophages from naive C57Bl/6 mice were stimulated with different concentrations Zymosan or *C. Albicans* for 16 hours (n=5). Levels of IL-1β, IL-6, IL-10 and TNF-α were determined by Luminex in the supernatants. Results are mean ± SEM.
(TIF)
