# Supplementary figures and images for: Exposure to Candida albicans Polarizes a T-Cell Driven Arthritis Model towards Th17 Responses, Resulting in a More Destructive Arthritis
Source: PLoS One. 2012 Jun 12;7(6):e38889. doi: 10.1371/journal.pone.0038889 (PMC3373564; doi:10.1371/journal.pone.0038889)

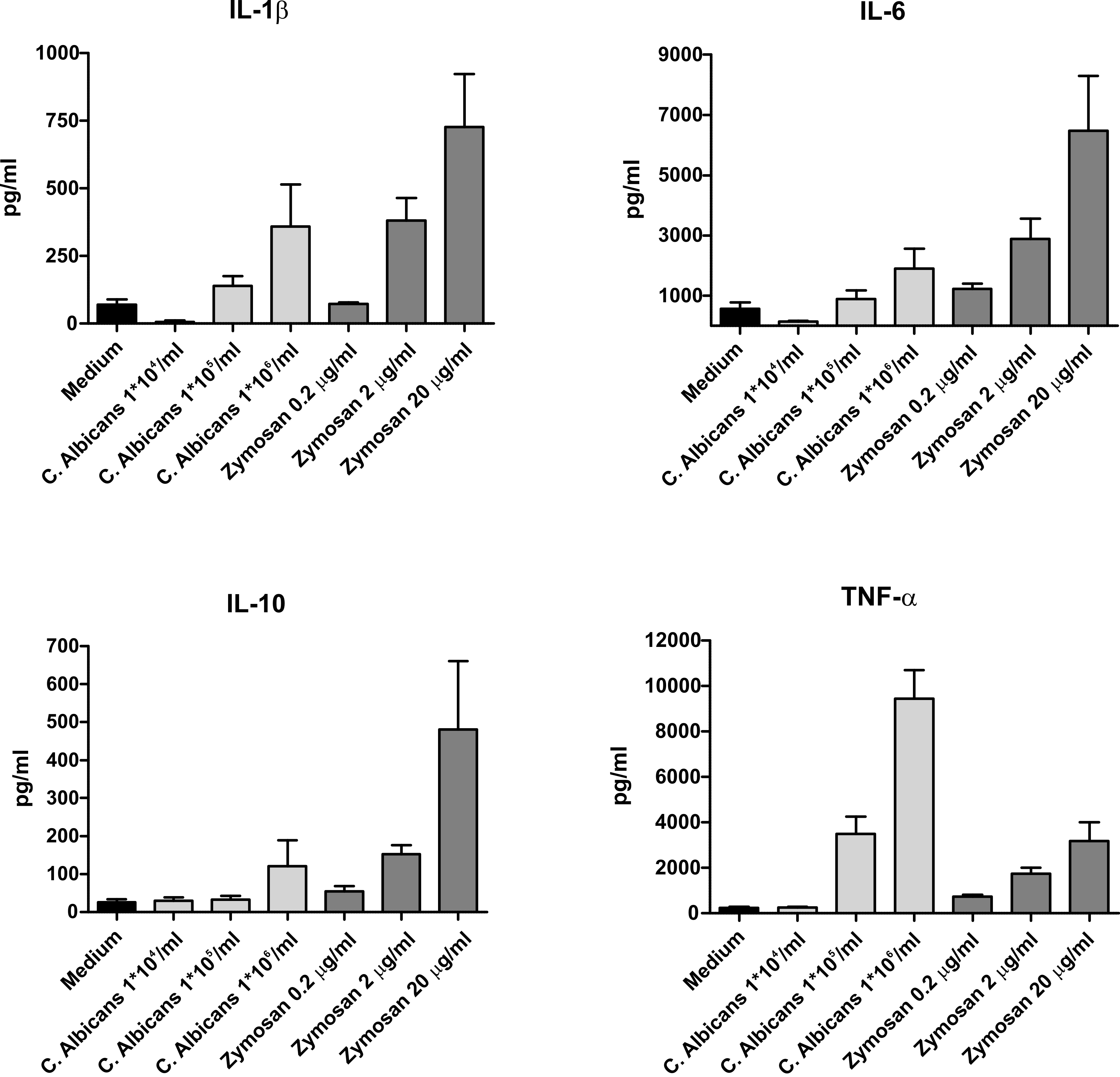

Supplement: Figure S1 — Candida albicans and Zymosan A induce a different cytokine profile in vitro. 1*105 peritoneal macrophages from naive C57Bl/6 mice were stimulated with different concentrations Zymosan or C. Albicans for 16 hours (n=5). Levels of IL-1β, IL-6, IL-10 and TNF-α were determined by Luminex in the supernatants. Results are mean ± SEM. (TIF) [file pone.0038889.s001.tif]
